# Down-regulation of lncRNA MALAT1 alleviates vascular lesion and vascular remodeling of rats with hypertension

**DOI:** 10.18632/aging.102113

**Published:** 2019-07-25

**Authors:** Yu-Zeng Xue, Zhi-Juan Li, Wei-Tao Liu, Jin-Jiao Shan, Lei Wang, Qian Su

**Affiliations:** 1 Cardiology Department, Liaocheng People’s Hospital, Liaocheng 252000, Shandong Province, PR.China; 2 Cardiovascular Department, The First Affiliated Hospital, and College of Clinical Medicine of Henan University of Science and Technology, Luoyang 471003, Henan Province, PR.China; 3 Cardiovascular Department, Minhang Hospital, Fudan University, Shanghai 201100, PR.China

**Keywords:** lncRNA MALAT1, Notch-1, hypertension, vascular lesion, vascular remodeling

## Abstract

Objective: Recently, the effect of long non-coding RNAs (lncRNAs) in hypertension (HTN) has been identified. This study aims to explore the expression of lncRNA metastasis-associated lung adenocarcinoma transcript 1 (MALAT1) in HTN and its role in vascular lesion and remodeling of HTN rats.

Results: LncRNA MALAT1 expression was up-regulated in HTN patients, and lncRNA MALAT1 could be an effective index of HTN diagnosis. Down-regulated MALAT1 and inhibited Notch-1 could reduce relative factor expression, including inflammation-related factors, endothelial function-related factors and oxidative stress-related factors, and inhibit apoptosis of aortic endothelial cells of HTN rats.

Methods: LncRNA MALAT1 expression in HTN patients and healthy controls was detected by reverse transcription quantitative polymerase chain reaction (RT-qPCR). Angiotensin II (Ang II)-induced HTN rat models were injected with MALAT1-siRNA, empty lentivirus vector, Notch pathway inhibitor (DAPT) and dimethyl sulphoxide (DMSO) via caudal vein. After three-week treatment, changes of blood pressure, inflammatory factor levels, endothelial function-related factors, oxidative stress indices and apoptosis of vascular endothelial cells were determined by a series of assays.

Conclusion: This study revealed that down-regulated lncRNA MALAT1 could alleviate the vascular lesion and remodeling of HTN rats, the mechanism may be related to the inhibited activation of Notch signaling pathway.

## INTRODUCTION

Hypertension (HTN) is the biggest cause of death all over the world, according to the statistic data, there were a third of adults, approximately 1 billion people in the world that influenced by hypertension, the date of which would enhanced to 1.6 billion by 2025 [1]. The multiple risk factors of HTN could be grouped into two types: congenital factors such as preterm birth [2] and Sasang constitutional types [3], acquired factors including sleep-disordered breathing [4], loneliness in later life [5] and sarcopenic obesity [6]. According to a study which was designed to investigate diagnosis and management of HTN, there generally appeared no obvious symptomatology in HTN, and about half of HTN patients remain undiagnosed [7]. Recently, a number of long non-coding RNAs (lncRNAs) have been identified and characterized in human diseases, and there were many extant studies have unraveled that lncRNAs were implicated in the development of HTN, such as lncRNA NONHSAT073641 [8], lncRNA sONE [9] and lncRNA TUG1 [10]. Nevertheless, the relation between lncRNA metastasis-associated lung adenocarcinoma transcript 1(MALAT1) and HTN has not been studied yet.

As one of the first found lncRNAs which were associated with cancers, MALAT1 was extensively expressed in human tissues [11], and the role of MALAT1 in human diseases such as nasopharyngeal carcinoma [12] and aggressive renal cell carcinoma [13] has been confirmed. Moreover, Zhuo *et al*. have elucidated in their study that MALAT1 was overexpressed in pulmonary arterial hypertension (PAH) [14]. In addition, Notch-1 is one of the four receptors of Notch signaling pathway, which has been proved to be correlated with tumor progression [15]. The relation between Notch-1 and HTN has been demonstrated in a recent study that Notch-1 increased proliferation of human pulmonary arterial endothelial cells (hPAECs) in pulmonary arterial hypertension and inhibition of Notch signaling could decrease proliferation and migration of hPAECs [16]. Furthermore, a study has demonstrated that MALAT1 knockdown could enhance chemosensitivity of ovarian cancer cells to cisplatin through inhibiting the Notch-1 signaling pathway [17]. However, the relation among MALAT1, Notch-1 and HTN has not been studied yet, hence, this study was conducted to focus on the capacity of MALAT1 and Notch-1 in HTN, and we speculated that down-regulated lncRNA MALAT1 could alleviate the vascular lesion and remodeling of HTN rats, the mechanism may be related to the inactivation of Notch signaling pathway.

## RESULTS

### MALAT1 expression was up-regulated in HTN patients

The comparison of baseline characteristics between HTN patients and healthy people indicated that there was no obvious difference in age, gender, body mass index (BMI), fasting blood glucose (FBG) and blood urea nitrogen (BUN) between HTN patients and healthy people (*P* > 0.05); while the difference in high-density lipoprotein cholesterol (HDL-C) and low-density lipoprotein cholesterol (LDL-C) was evident between HTN patients and healthy people (Table 1, *P <* 0.05).

**Table 1:** Comparison of baseline characteristics between HTN patients and healthy people.

**Indexes**	**HTN patients (n = 30)**	**Healthy people (n = 30)**	***P***
Age (years old)	57.30 ± 8.11	58.53 ± 8.02	0.557
Male/female	17/13	16/14	0.795
History of HTN (≥ 10 years/ < 10 years)	8/22	/	/
Antihypertensive drug (yes/no)	13/17	/	/
BMI (Kg/m^2^)	25.39 ± 4.08	24.71 ± 3.57	0.495
FBG (mmol/L)	5.61 ± 0.59	5.49 ± 0.76	0.497
HDL-C (mmol/L)	1.02 ± 0.24	1.28 ± 0.26	*<* 0.001
LDL-C (mmol/L)	2.67 ± 0.63	2.03 ± 0.58	*<* 0.001
BUN (mmol/L)	5.06 ± 0.44	4.85 ± 0.71	0.174

Notes: HTN, hypertension; BMI, body mass index; FBG, fasting blood glucose; HDL-C, high-density lipoprotein cholesterol; LDL-C, low-density lipoprotein cholesterol; BUN, blood urea nitrogen. The enumeration data were expressed as percentage form and analyzed by chi-square test, and the measurement data were expressed as mean ± standard deviation, comparisons between two groups were conducted by *t*-test.

**Figure 1:**
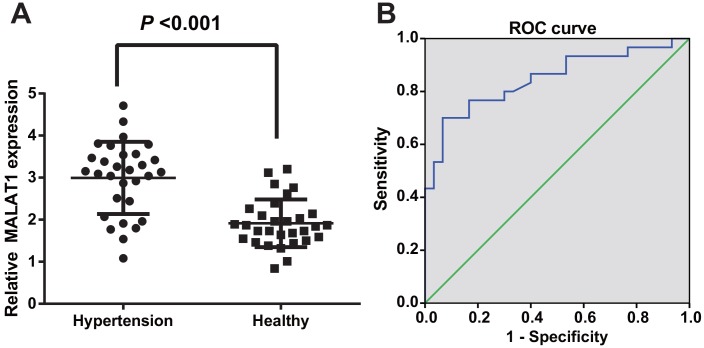
**LncRNA MALAT1 was up-regulated in HTN patients.** (**A**) expression of lncRNA MALAT1 in healthy controls and HTN patients; (**B**) ROC curve represented for the efficiency of lncRNA MALAT1 expression to the diagnosis of HTN patients. n = 30, data were expressed as mean ± standard deviation.

RT-qPCR was employed to evaluate lncRNA MALAT1 expression in HTN patients and healthy people to observe the level of lncRNA MALAT1 in HTN patients, the results of which showed that the lncRNA MALAT1 expression of HTN patients was up-regulated compared with healthy controls, suggesting that MALAT1 was highly expressed in the serum of HTN patients (*P <* 0.05; Figure 1A).

To further identify the diagnostic efficiency of MALAT1 expression in HTN patients, the diagnostic relation between MALAT1 and HTN was assessed by ROC curve, outcome of which unraveled that the AUC was 0.847 and 50% confidence interval (CI) was 0.746-0.947 (*P* < 0.001); sensitivity and specificity were 0.700 and 0.933, implying that lncRNA MALAT1 may be an effective biomarker in the diagnosis of HTN (Figure 1B).

### LncRNA MALAT1 and Notch-1 were highly expressed in vascular tissues of rats with HTN

**Figure 2:**
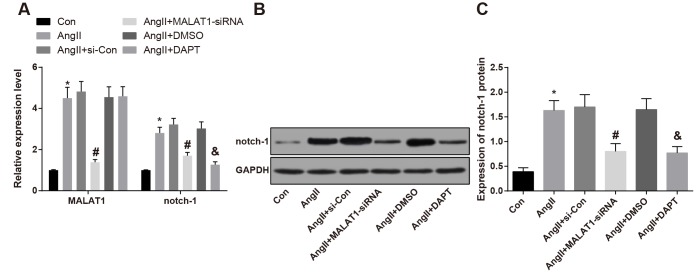
**Expression of lncRNA MALAT1 and Notch-1 was elevated in vascular tissues of HTN rats.** (**A**) expression of of lncRNA MALAT1 and Notch-1 in thoracic aorta vascular tissues detected by RT-qPCR; (**B**) protein band of Notch-1 in thoracic aorta vascular tissues; (**C**) expression of Notch-1 in thoracic aorta vascular tissues detected by western blot analysis; * *P* < 0.05 *vs* the Con group; # *P* < 0.05 *vs* the AngII + si-Con group; & *P* < 0.05 *vs* the AngII + DMSO group; n = 10, data were expressed as mean ± standard deviation; one-way ANOVA was used for analyzing data, pairwise comparison was analyzed by Tukey’s post hoc test.

After the HTN rats were treated with down-regulated MALAT1 vector and Notch pathway inhibitor (DAPT), the changes of expression of lncRNA MALAT1 and Notch-1 in rat’s vascular tissues were measured by RT-qPCR and western blot analysis, the results indicated that the lncRNA MALAT1 and Notch-1 expression in the AngII group was up-regulated compared with the Con group (*P <* 0.05), expressed for the high expression of lncRNA MALAT1 and Notch-1 in HTN vascular tissues. MALAT1 and Notch-1 expression in the AngII + MALAT1-siRNA group down-regulated relative to the AngII + si-Con group (*P <* 0.05), and expression of Notch-1 in the AngII + DAPT group down-regulated in comparison to the AngII + DMSO group (*P <* 0.05. There was no significant difference of lncRNA MALAT1 and Notch-1 among the AngII group, AngII + si-Con group and AngII + DMSO group (Figure 2A–2C, *P* > 0.05), indicating that MALAT1-siRNA vector and Notch-1 pathway inhibitor could suppress the expression of MALAT1 and Notch-1.

### Down-regulated MALAT1 and inhibited Notch-1 decrease blood pressure level of rats with HTN

**Figure 3:**
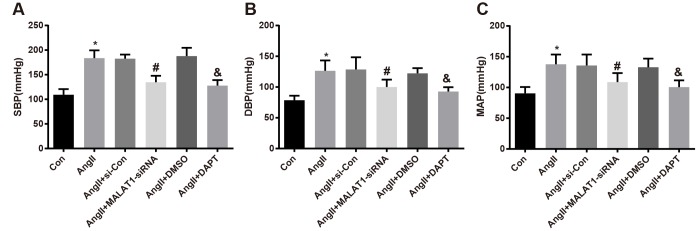
**Down-regulated MALAT1 and inhibited Notch-1 decline blood pressure level of rats with HTN.** (**A**) comparison of SBP of rats; (**B**) comparison of DBP of rats; (**C**) comparison of MAP of rats; * *P* < 0.05 *vs* the Con group; # *P* < 0.05 *vs* the AngII + si-Con group; & *P* < 0.05 *vs* the AngII + DMSO group; n = 10, data were expressed as mean ± standard deviation; one-way ANOVA was used for analyzing data, pairwise comparison was analyzed by Tukey’s post hoc test.

In order to investigate the effects of repressed MALAT1 and inhibited Notch-1 signaling pathway on the blood pressure level of HTN rats, the blood pressure of rats in each group was determined by a tail-artery blood pressure-measuring instrument, the results unraveled that the levels of SBP, DBP and MAP of rats in the AngII group significantly increased compared with the Con group (all *P <* 0.05). The levels of SBP, DBP and MAP of rats in the AngII + MALAT1-siRNA group significantly decreased in comparison to the AngII + si-Con group (all *P <* 0.05), and the levels of which in the AngII + DAPT group also significantly decreased relative to the AngII + DMSO group (all *P <* 0.05). No significant difference was found of SBP, DBP and MAP among the AngII group, AngII + si-Con group and AngII + DMSO group (Figure 3A–3C, all *P* > 0.05), suggesting that down-regulated MALAT1 and inhibited Notch-1 could decrease blood pressure level of HTN rats.

### Down-regulated MALAT1 and inhibited Notch-1 repress inflammation-related factor expression in rats with HTN

**Figure 4:**
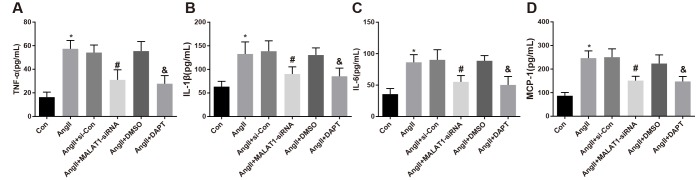
**Down-regulated MALAT1 and inhibited Notch-1 repress inflammation-related factors expression in rats with HTN.** (**A**) comparison of TNF-α levels in serum of rats; (**B**) comparison of IL-1β levels in serum of rats; (**C**) comparison of IL-6 levels in serum of rats; (**D**) comparison of MCP-1 levels in serum of rats; * *P* < 0.05 *vs* the Con group; # *P* < 0.05 *vs* the AngII + si-Con group; & *P* < 0.05 *vs* the AngII + DMSO group; n = 10, data were expressed as mean ± standard deviation; one-way ANOVA was used for analyzing data, pairwise comparison was analyzed by Tukey’s post hoc test.

To explore the role of down-regulated MALAT1 and inhibited Notch-1 signaling pathway in the levels of inflammation-related factors, ELISA was adopted to measure the expression of inflammation-related factors in serum of rats in each group, the results suggested that the levels of TNF-α, IL-1β, IL-6 and MCP-1 of rats in the AngII group significantly increased in contrast to the Con group (all *P <* 0.05). The levels of TNF-α, IL-1β, IL-6 and MCP-1 of rats in the AngII + MALAT1-siRNA group significantly decreased in comparison to the AngII + si-Con group (all *P <* 0.05), and the levels of which of the AngII + DAPT group markedly decreased compared with the AngII + DMSO group (all *P <* 0.05). No obvious difference in TNF-α, IL-1β, IL-6 and MCP-1 was observed among the AngII group, AngII + si-Con group and AngII + DMSO group (Figure 4A–4D, all *P* > 0.05), implying that down-regulated MALAT1 and inhibited Notch-1 signaling pathway could alleviate the inflammatory reaction of rats in each group.

### Down-regulated MALAT1 and inhibited Notch-1 reduce endothelial function-related factors expression in rats with HTN

**Figure 5:**
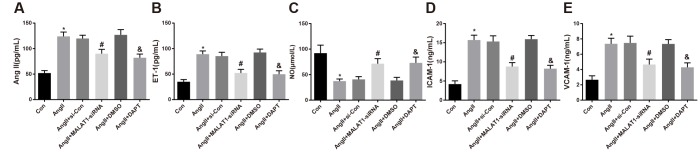
**Down-regulated MALAT1 and inhibited Notch-1 reduce endothelial function-related factors expression in rats with HTN.** (**A**) AngII in serum of rats; (**B**) ET-1 in serum of rats; (**C**) NO in serum of rats; (**D**) ICAM-1 in serum of rats; (**E**) VCAM-1 in serum of rats; * *P* < 0.05 *vs* the Con group; # *P* < 0.05 *vs* the AngII + si-Con group; & *P* < 0.05 *vs* the AngII + DMSO group; n = 10, data were expressed as mean ± standard deviation; one-way ANOVA was used for analyzing data, pairwise comparison was analyzed using Tukey’s post hoc test.

To explore the role of down-regulated MALAT1 and inhibited Notch-1 signaling pathway in the levels of endothelial function-related factors, ELISA was adopted to evaluate the levels of endothelial function-related factors in serum of the rats. The results revealed that the levels of AngII, ET-1, ICAM-1 and VCAM-1 of rats in the AngII group remarkably increased compared with the Con group, while the level of NO significantly decreased (all *P <* 0.05). The levels of AngII, ET-1, ICAM-1 and VCAM-1 of rats in the AngII + MALAT1-siRNA group decreased contrasted with the AngII + si-Con group (all *P <* 0.05), while the level of NO increased (*P <* 0.05). The levels of AngII, ET-1, ICAM-1 and VCAM-1 of rats in the AngII + DAPT group decreased compared with the AngII + DMSO group (all *P <* 0.05), while the level of NO increased (*P <* 0.05). There was no significant difference in AngII, ET-1, ICAM-1, VCAM-1 and NO levels among the AngII group, AngII + si-Con group and AngII + DMSO group (Figure 5A–5E, all *P* > 0.05), indicating that down-regulated MALAT1 and inhibited Notch-1 signaling pathway could ameliorate the levels of endothelial function-related factors of rats in each group.

### Down-regulated MALAT1 and inhibited Notch-1 decline oxidative stress-related factors expression in rats with HTN

**Figure 6:**
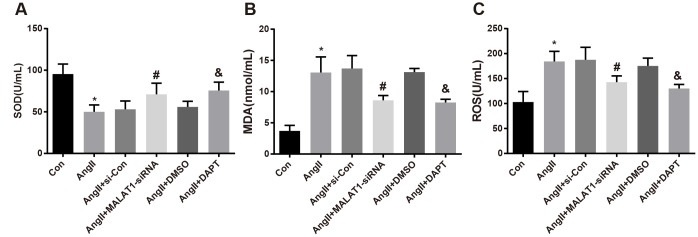
**Down-regulated MALAT1 and inhibited Notch-1 decline oxidative stress-related factors expression in rats with HTN.** (**A**) comparison of SOD in serum of rats; (**B**) comparison of MDA in serum of rats; (**C**) comparison of ROS in serum of rats; * *P* < 0.05 *vs* the Con group; # *P* < 0.05 *vs* the AngII + si-Con group; & *P* < 0.05 *vs* the AngII + DMSO group; n = 10, data were expressed as mean ± standard deviation; one-way ANOVA was used for analyzing data, pairwise comparison was analyzed by Tukey’s post hoc test.

To investigate the role of down-regulated MALAT1 and inhibited Notch-1 signaling pathway in the expression of oxidative stress-related factors, oxidative stress-related factors expression in serum of the rats was measured, the outcomes of which revealed that the levels of MDA and ROS of rats in the AngII group increased in comparison to the Con group, while the level of SOD reduced (all *P <* 0.05). The levels of MDA and ROS of rats in the AngII + MALAT1-siRNA group significantly decreased contrasted with the AngII + si-Con group, while the level of SOD enhanced (all *P <* 0.05). The levels of MDA and ROS of rats in the AngII + DAPT group attenuated in contrast to the AngII + DMSO, while the level of SOD elevated (all *P <* 0.05). No significant difference in the levels of MDA, ROS and SOD was observed among the AngII group, AngII + si-Con group and AngII + DMSO group (Figure 6A–6C, all *P* > 0.05), suggesting that down-regulated MALAT1 and inhibited Notch-1 signaling pathway could improve oxidative stress reaction of rats in each group.

### Down-regulated MALAT1 and inhibited Notch-1 alleviate pathological lesion of thoracic aorta

**Figure 7:**
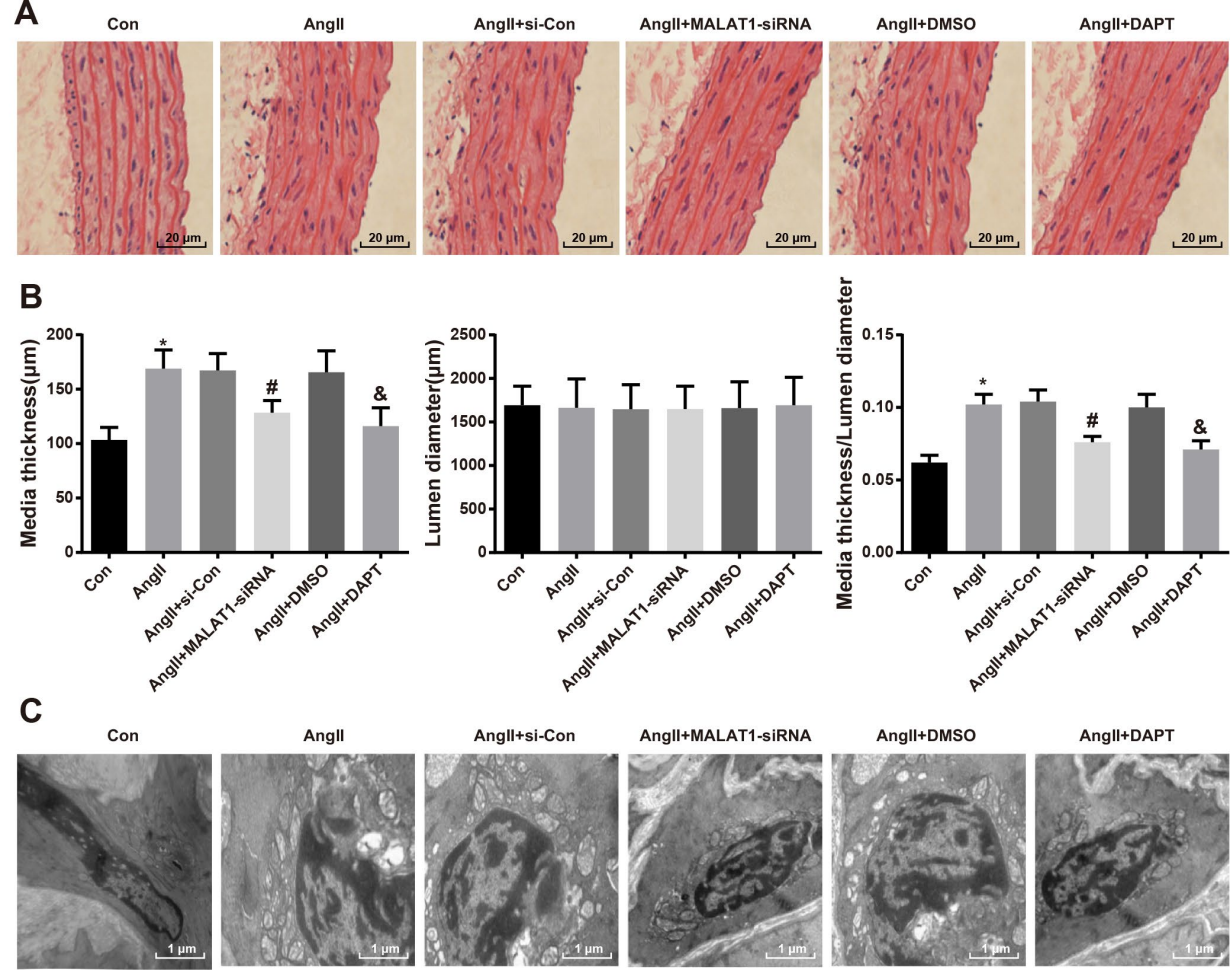
**Down-regulated MALAT1 and inhibited Notch-1 relieve pathological lesion of thoracic aorta.** (**A**) thoracic aorta tissues of rats were observed using HE staining (× 500, scale bar: 20 μm); (**B**) medial thickness and lumen diameter in thoracic aorta of rats and their ratio; (**C**) ultrastructure of thoracic aorta of rats were observed by a transmission electron microscopy (× 10000, scale bar: 1 μm). * *P* < 0.05 *vs* the Con group; # *P* < 0.05 *vs* the AngII + si-Con group; & *P* < 0.05 *vs* the AngII + DMSO group; n = 10, data were expressed as mean ± standard deviation; one-way ANOVA was used for analyzing data, pairwise comparison was analyzed by Tukey’s post hoc test.

To explore the role of down-regulated MALAT1 and inhibited Notch-1 signaling pathway in pathological lesion of thoracic aorta, HE staining was employed to assess the pathological changes of thoracic aorta of rats in each group, the results of which indicated that the morphology of aorta in rats of the Con group was normal, with clear boundaries, smooth endangium and without hypertrophy, cell adhesion and cell migration; the rats of AngII group, AngII + si-Con group and AngII + DMSO group presented with aorta thickness, rough endangium and disordered structure, increase and migration of smooth muscle cells were visible, and there were derangement, abscission and fracture in the elastic fibers; the hypertrophy of arterial walls was relieved, the increase and migration of smooth muscle cells were decreased and the disorder as well as fracture of elastic fibers were alleviated in the AngII + MALAT1-siRNA and the AngII + DAPT groups (Figure 7A).

As it was shown in the quantitative result of the image analyzer, the medial thickness and the ratio of medial thickness and lumen diameter of rats in the AngII group enhanced in comparison to the Con group (*P <* 0.05). The medial thickness and the ratio of medial thickness and lumen diameter of rats in the AngII + MALAT1-siRNA group declined relative to the AngII + si-Con group (*P <* 0.05). The medial thickness and the ratio of medial thickness and lumen diameter of rats in the AngII + DAPT group decreased in contrast to the AngII + DMSO group (*P <* 0.05). There was no obvious difference of lumen diameter among the AngII group, AngII + si-Con group, AngII + MALAT1-siRNA group, AngII + DMSO group and AngII + DAPT group (all *P* > 0.05), and there was also no significant difference of medial thickness, lumen diameter and the ratio of medial thickness and lumen diameter among the AngII group, AngII + si-Con group and AngII + DMSO group (Figure 7B, *P* > 0.05).

Transmission electronic microscope was used to observe the ultrastructure of thoracic aorta of rats in each group, the outcomes of which implied that there were normal ultrastructure of thoracic aorta, smooth endangium and normal morphology of endothelial cells and smooth muscle cells in the rats of the Con group. While there were aorta thickness, rough endangium, separation and breakage of internal elastic lamina and disorder and hypertrophy of vascular smooth muscle cells in tunica media in the AngII group, AngII + si-Con group and AngII + DMSO group, with the hypertrophy of collagenous fibers, there appeared serious lesion and an increasing number of intracellular organelles. There were smooth endangium, complete structure, obscure hypertrophy of smooth muscle cells, continuous elastic fibrous membranes in tunica media, alleviated lesion of endothelial cells and decrease of intracellular organelles in the AngII + MALAT1-siRNA and the AngII + DAPT groups (Figure 7C). These results implied that down-regulated MALAT1 and inhibited Notch-1 alleviate pathological lesion of thoracic aorta of HTN rats

### Down-regulated MALAT1 and inhibited Notch-1 alleviate vascular fibrosis

**Figure 8:**
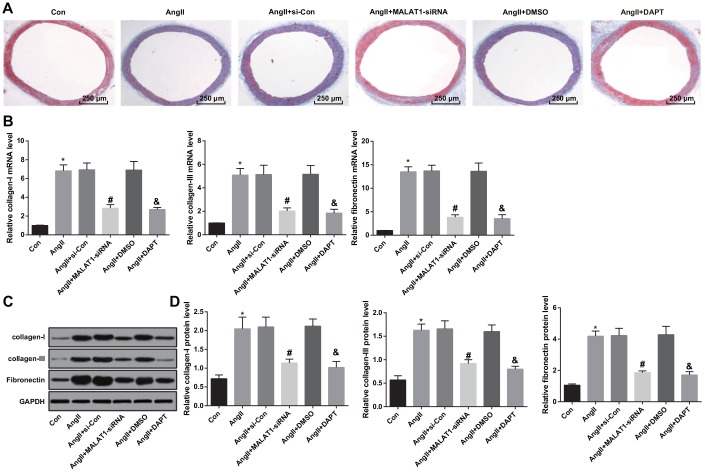
**Down-regulated MALAT1 and inhibited Notch-1 alleviate vascular fibrosis.** (**A**) thoracic aorta fibrosis was observed using Masson staining (× 40, scale bar: 250 μm); (**B**) expression of collagen-I, collagen-III and Fibronectin mRNA in thoracic aorta tissues of rats detected by RT-qPCR; (**C**) protein bands of collagen-I, collagen-III and Fibronectin in thoracic aorta tissues of rats; (**D**) expression of collagen-I, collagen-III and Fibronectin in thoracic aorta tissues of rats detected by western blot analysis; * *P* < 0.05 *vs* the Con group; # *P* < 0.05 *vs* the AngII + si-Con group; & *P* < 0.05 *vs* the AngII + DMSO group; n = 10, data were expressed as mean ± standard deviation; one-way ANOVA was used for analyzing data, pairwise comparison was analyzed by Tukey’s post hoc test.

Masson staining was adopted in order to investigate the effects of down-regulated MALAT1 and inhibited Notch-1 on vascular fibrosis of thoracic aorta of HTN rats, the result of Masson staining suggested that there was obvious and complete structure of thoracic aorta, slender and uniform distribution in the rats of the Con group. While there was distribution disorder of collagenous fibers in aorta, increase of blue collagen deposition and severe vascular fibrosis in the AngII group, AngII + si-Con group and AngII + DMSO group. There was alleviation of distribution disorder of collagenous fibers in aorta, a small amount of blue collagen and mitigation of vascular fibrosis in the AngII + MALAT1-siRNA group and the AngII + DAPT group (Figure 8A).

The changes of collagen-I, collagen-III and Fibronectin expression in thoracic aorta were measured by RT-qPCR and western blot analysis, the results implied that the expression of collagen-I, collagen-III and Fibronectin in the AngII group were up-regulated compared with the Con group (all *P <* 0.05). The expression of collagen-I, collagen-III and Fibronectin in the AngII + MALAT1-siRNA group were down-regulated in contrast to the AngII + si-Con group (all *P <* 0.05). The expression of collagen-I, collagen-III and Fibronectin in the AngII + DAPT group were down-regulated in comparison to the AngII + DMSO group (all *P <* 0.05). There was no obvious difference of collagen-I, collagen-III and Fibronectin expression among the AngII group, AngII + si-Con group and AngII + DMSO group (Figure 8B–8D, all *P* > 0.05), indicating that down-regulated MALAT1 and inhibited Notch-1 signaling pathway could alleviate vascular fibrosis of thoracic aorta of HTN rats.

### Down-regulated MALAT1 and inhibited Notch-1 suppress apoptosis of aortic endothelial cells

**Figure 9:**
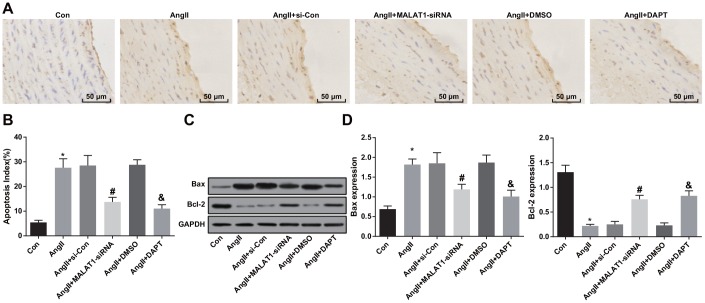
**Down-regulated MALAT1 and inhibited Notch-1 suppress apoptosis of aortic endothelial cells.** (**A**) endothelial cells of thoracic aorta were observed by TUNEL staining (× 200, scale bar: 50 μm); (**B**) apoptosis index of endothelial cells in thoracic aorta; (**C**) protein bands of Bax and Bcl-2 of thoracic aorta tissues; (**D**) expression of Bax and Bcl-2 in thoracic aorta; * *P* < 0.05 *vs* the Con group; # *P* < 0.05 *vs* the AngII + si-Con group; & *P* < 0.05 *vs* the AngII + DMSO group; n = 10, data were expressed as mean ± standard deviation; one-way ANOVA was used for analyzing data, pairwise comparison was analyzed by Tukey’s post hoc test.

To observe the impacts of down-regulated MALAT1 and inhibited Notch-1 signaling pathway on apoptosis of aortic endothelial cells of HTN rats, TUNEL staining was employed to assess the apoptosis of aortic endothelial cells of HTN rats, and the results indicated that the apoptosis index of endothelial cells of rats in the AngII group increased compared with the Con group (*P <* 0.05). The apoptosis index of endothelial cells in the AngII + MALAT1-siRNA group reduced relative to the AngII + si-Con group (*P <* 0.05). The apoptosis index of endothelial cells in the AngII + DAPT group abated in contrast to the AngII + DMSO group (*P <* 0.05). There was no obvious difference of apoptosis index of endothelial cells among the AngII group, AngII + si-Con group and AngII + DMSO group (Figure 9A–9B, *P* > 0.05).

The protein expression of Bax and Bcl-2 in thoracic aorta of rats were measured using western blot analysis, and the results suggested that the protein expression of Bax increased and the protein expression of Bcl-2 decreased in the AngII group compared with the Con group (both *P <* 0.05). The protein expression of Bax decreased and the protein expression of Bcl-2 increased in the AngII + MALAT1-siRNA group in comparison to the AngII + si-Con group (*P <* 0.05). The protein expression of Bax decreased and the protein expression of Bcl-2 increased in the AngII + DAPT group compared with the AngII + DMSO group (*P <* 0.05). There was no significant difference of protein expression of Bax and Bcl-2 among the AngII group, AngII + si-Con group and AngII + DMSO group (Figure 9C–9D, *P* > 0.05), suggesting that down-regulated MALAT1 and inhibited Notch-1 signaling pathway could decelerate apoptosis of aortic endothelial cells of HTN rats.

## DISCUSSION

HTN is the commonest chronic disease, which was diagnosed in one third adults, whether in developed countries or developing countries [18]. It has been clarified that lncRNAs play a key part in the progression of many intricate diseases [19]. Moreover, there were several researches implied that the mechanism of lncRNA MALAT1 might be associated with human diseases, such as MALAT1 could accelerate cell proliferation in gastric cancer via modulating SF2/ASF [11], adjust cancer stem cell (CSC) activity and radioresistance [12] and attenuate the development of renal cancer cells [20]. Nevertheless, there is little known about lncRNA MALAT1 as well as Notch-1 in progression of HTN. Consequently, this study was determined to explore the impact of lncRNA MALAT1 and Notch-1 on HTN, and we have found in this study that down-regulated lncRNA MALAT1 and Notch-1 could alleviate the vascular lesion and remodeling of HTN rats.

Among the vital results in our research, one of them suggested that lncRNA MALAT1 was highly expressed in HTN, implying that there was aberrant expression of lncRNA MALAT1 in HTN. This dysregulation of lncRNA MALAT1 has also been identified in other human diseases, such as gastric cancer [11] and pulmonary arterial hypertension [14]. Moreover, we have found in the results that Notch-1 also performed an up-regulation in HTN, which was similar to that in chronic lymphocytic leukemia [21] and T lymphocytes [22]. Additionally, the results in this study have demonstrated that there was a negative relationship between lncRNA MALAT1 and Notch-1, and this result was confirmed by a recent study [17]. These studies contributed to the recognition of the roles of lncRNA MALAT1 and Notch-1 and their molecular mechanisms in HTN.

Another important finding in our study was that the expression of inflammation-related factors, including TNF-α, IL-1β, IL-6 and MCP-1, could be suppressed by silencing lncRNA MALAT1 and Notch-1. Similar to this result, evidence has been provided in a recent study which proved that knockdown of MALAT1 was able to inhibit the levels of TNF-α, IL-1β and IL-6 [23]. As to the correlation among Notch-1 and inflammation-related factors, according to the outcomes of a recent research, expression of IL-1, IL-6 and TNF-α could be abated by siRNA against Notch1 [24]. Besides, the levels of endothelial function-related factors such as AngII, ET-1, ICAM-1 and VCAM-1 were attenuated by inhibiting lncRNA MALAT1 and Notch-1. It is in accordance with our finding that a previous research has also verified that ICAM-1 was implicated in Notch-1 signaling pathway in breast cancer [25]. The effect of MALAT1 knockdown on endothelial cell function was evident in an available literature that silencing of MALAT1 may accelerate the migration of endothelial cells [26]. Another essential finding of this study has revealed that down-regulated lncRNA MALAT1 and inhibited Notch-1 could reduce the levels of oxidative stress-related factors and suppressed apoptosis of aortic endothelial cells. In line with this result, the role of MALAT1 in inhibiting ROS, which is one of the oxidative stress-related factors has been illustrated [27]. Similarly, Guoping Wang *et al*. have proved that lncRNA MALAT1 could suppress the apoptosis of human cerebrovascular endothelial cells via reducing PI3K activity [28]. An analogous result could also be found in another research which has identified that down-regulation of lncRNA MALAT1 could protect cardiomyocytes from apoptosis [29]. One more vital outcome performed in our research implied that down-regulated MALAT1 and inhibited Notch-1 could alleviate vascular fibrosis, which has also been illuminated by Biao Hu *et al*. [30]. As to the correlation between Notch-1 and endothelial cell apoptosis, a recent study has demonstrated that Notch-1 could inhibit endothelial cell apoptosis in pulmonary arterial hypertension via Bcl-2 and Survivin [16]. All of these data have provided evidence to further prove the mechanism and function of lncRNA MALAT1 and Notch-1 in human diseases.

In conclusion, our study provides evidence that up-regulated lncRNA MALAT1 was implicated in regulation of HTN, what’s more, down-regulated lncRNA MALAT1 and inhibited Notch-1 could improve endothelial function, reduced mRNA expression of relative factors, including inflammation-related factors, endothelial function-related factors and oxidative stress-related factors of rats with HTN and restrained apoptosis of aortic endothelial cells, which provided a novel way of HTN therapy. However, further study is required to better elucidate the function mechanisms of lncRNA MALAT1 and Notch-1 in the progression of HTN.

## MATERIALS AND METHODS

### Ethics statement

Written informed consents were obtained from all patients prior to the study. The protocols of this study were approved by the Ethic Committee of Minhang Hospital, Fudan University and based on the ethical principles for medical research involving human subjects of the Helsinki Declaration. Animal experiments were strictly in accordance with the Guide to the Management and Use of Laboratory Animals issued by the National Institutes of Health. The protocol of animal experiments was approved by the Institutional Animal Care and Use Committee of Minhang Hospital, Fudan University.

### Study subjects

A total of 30 samples of venous blood from HTN patients (aging 43–72 years old with a mean age of 57.30 ± 8.11 years) who received resection in Minhang Hospital, Fudan University from September 2017 to February 2019 were collected, 17 males and 13 females. Thirty samples from healthy people (aging 47–74 years old with a mean age of 58.53 ± 8.02 years) were collected as control group, 16 males and 14 females. The samples were supernatant from each patient and healthy people after centrifugation (5 mL venous blood). All the patients were selected with certain diagnostic standard of HTN [31], the patients were older than 45 years and were excluded from this study if they had diabetes, cardiovascular and cerebrovascular diseases such as coronary heart disease, or if they had malignant tumor; or women in pregnant stage or breast-feed stage.

### Preparation of lentivirus vector

MALAT1 small interfering RNA (siRNA) sequence (5′-GTGACTTAAACAGCTTAAATT-3′) was cloned into pENTR vector (Invitrogen, Carlsbad, CA, USA). The vector and lentivirus packaging plasmid (GenePharma Co., Ltd., Shanghai, China) were transfected into 293T cells according to the instruction of Lipofectamine 2000 (Invitrogen, Carlsbad, CA, USA). Supernatant containing lentivirus were concentrated and purified by filtering and centrifuging after 48–72 h transfection, and the high-titer lentivirus fluid was synthetized by Genechem Co., Ltd. (Shanghai, China). The concentrate was preserved in a -80°C refrigerator. The number of fluorescent cells was counted under a fluorescence microscopy, and the titer was measured as 3 × 10^8^ TU/mL [32].

### Experimental animals, model preparation and grouping

Seventy male Sprague Dawley (SD) rats (aging 10 weeks, weighing 250–300 g) were purchased from Qiming Biotechnology Co., Ltd., (Shanghai, China). All the rats were adaptively fed for one week, at room temperature 20–25°C, humidity 55–60% with general day/night cycle and free access to food and water in clean environment, which was kept quiet.

Preparation of HTN models: 60 SD rats were anaesthetized by ketamine (100 mg/kg) and xylazine (10 mg/kg). According to a relative literature [33], the rats were implanted with pressure-regulating micro osmotic pump (Alzet, NY, USA) and slowly injected with angiotensionII (AngII) (200 ng/kg/min) for 2 weeks, which was purchased from Kemin Biotechnology Co., Ltd., (Shanghai, China). The blood pressure and weight of the rats were detected and the changes of blood pressure were recorded regularly in the following 1 week, the models were considered to be constructed successfully when the blood pressure was higher than 160 mm Hg. Fifty rats from the successful models were divided into HTN model (AngII) group, AngII + lentivirus vector (AngII + si-Con) group, AngII + MALAT1-siRNA group, AngII + Notch pathway inhibitor (AngII + 2,4-diamino-5-phenylthiazole (DAPT) group) and AngII + dimethyl sulfoxide (DMSO) group, 10 rats in each group; the other 10 rats was selected as control (Con) group.

Con group: the steps were in accordance with AngII group except for replacing AngII with equal normal saline; AngII group: establishment of HTN models were induced and constructed by AngII; AngII + MALAT1-siRNA group: establishment of HTN models were induced and constructed by AngII, there was 150 μL lentivirus containing MALAT1-siRNA was injected in tail vein of rats, transfection reagent was diluted to a titer of 1 × 10^8^ TU/mL and the rats were fed for 3 weeks; AngII + DAPT group: establishment of HTN models were induced and constructed by AngII and 5 mg/kg DAPT was injected in tail vein of rats every one day for 3 weeks; AngII + DMSO group: establishment of HTN models were induced and constructed by AngII and 5 mg/kg DMSO was injected in tail vein of rats every one day for 3 weeks. DAPT and DMSO were purchased from Biolab Technology Co., Ltd., (Beijing, China).

### Detection of blood pressure-related indexes

After three-week intervention treatment, rats were placed in the environment to be tested for 30 min, systolic blood pressure (SBP), diastolic blood pressure (DBP) and mean arterial pressure (MAP) were measured using a tail-artery blood pressure-measuring instrument (Softron Biotechnology Co., Ltd., Tokyo, Japan) by putting the instrument at the root of tail.

### Measurement of serum factors

After three-week intervention treatment, 5 mL blood of abdominal aorta collected from the rats was placed for 30 min, centrifuged for 10 min at 3000 r/min and the supernatant was adopted, enzyme-linked immunosorbent assay (ELISA) was used to evaluate the content of tumor necrosis factor-α(TNF-α), interleukin-1β (IL-1β), interleukin-6 (IL-6), monocyte chemotactic protein-1 (MCP-1), Ang II, intercellular adhesion molecule-1 (ICAM-1), vascular cell adhesion molecule 1 (VCAM-1) and endothelin-1 (ET-1) according to the instruction of the kits purchased from Beckman Coulter, Inc. (CA, USA). The content of nitric oxide (NO) was detected by nitric reductase method, the content of reactive oxygen species (ROS) was measured by colorimetry, the content of malondialdehyde (MDA) was evaluated by thiobarbituric acid and the content of superoxide dismutase (SOD) was detected by xanthine oxidase method, the reagent used for testing NO, ROS, MDA and SOD were all purchased from Jingmei Bioengineering Co., Ltd., (Shenzhen, China).

### Collection of tissue samples

The rats were euthanized by excessive sodium pentobarbital and the thoracic aorta was separated and washed by phosphate buffered solution (PBS) twice. A part of the thoracic aorta tissues were fixed in 4% paraformaldehyde for 24 h for the hematoxylin-eosin (HE) staining, Masson staining and detection of cell apoptosis, the other part was fixed in 2.5% glutaraldehyde for the observation of ultrastructure. Besides, some of the thoracic aorta tissues were preserved by liquid nitrogen in the frozen pipes for the measurement of mRNA and expression of relevant proteins.

### HE staining and ultrastructural observation of thoracic aorta

HE staining: thoracic aorta was fixed in 4% paraformaldehyde for 24 h, paraffin-embedded and sectioned. The sections were stained by hematoxylin and eosin for 10 min, differentiated by 1% hydrochloric alcohol for 10 s, then treated by 2% sodium bicarbonate for 10 s; disseminated in eosin for 3 min, dehydrated in ascending series of ethanol, cleared in xylene, mounted and captured. Meanwhile, thickness of tunicae media vasorum, lumen diameter and their ratio were evaluated.

Ultrastructural observation of thoracic aorta: thoracic aorta fixed in 2.5% glutaraldehyde was fixed again by 1% osmic acid and dehydrated by ethanol, sectioned by a thin-sliced cutting machine (Olympus, Tokyo, Japan), then double stained by 3% uranyl acetate-lead citrate, and the changes of ultrastructural observation of thoracic aorta were observed by a transmission electron microscopy (Hitathi Co., Ltd., Tokyo, Japan).

### Masson staining

The aorta vascular tissues were departed, sectioned and dewaxed according to the steps of HE staining. The tissue sections were dewaxed by xylene (3 times, 5 min/time) and soaked in graded ethanol (100%, 90%, 85%, 75%, each for 5 min). The sections were conducted with Masson staining according to the instruction of Masson kit (Service Biological Technology, Wuhan, China) and the changes of vascular fiber were observed (the blue part is deposition of collagen) under a light microscopy (Olympus, Tokyo, Japan). Image-Pro Plus software was used for image analysis.

### Terminal deoxynucleotidyl transferase-mediated dUTP nick end-labeling (TUNEL) staining

The aorta vascular tissues were fixed and made to paraffin sections, and the cell apoptosis of aortic endothelium was evaluated by TUNEL kit (Merck, Darmstadt, Hesse-Darmstadt, Germany) and observed by a laser scanning confocal microscopy (Olympus, Tokyo, Japan). Three sections of each group were observed and 5 fields of view were selected from each section. Apoptosis index of vascular endothelial cells = the number of vascular endothelial cells/the number of total cells × 100% and the mean was adopted.

### Reverse transcription quantitative polymerase chain reaction (RT-qPCR)

Trizol (Invitrogen, Carlsbad, CA, USA) assay was adopted to extract the total RNA of the specimens. MALAT1, Notch-1, Collagen-I, Collagen-III, fibronectin and glyceraldehyde phosphate dehydrogenase (GAPDH) primers were synthetized by Invitrogen Inc. (Carlsbad, California, USA) (Table 2), GAPDH was used as the internal reference. Then RNA was reversely transcripted to cDNA by PrimeScript RT kit according to the instructions. RT-qPCR was conducted by SYBR Green PCR Master Mix kit (Roche, Indianapolis, IN, USA) according to the instructions. The relative expression of the target genes was calculated by 2^-ΔΔCt^ method, △△Ct = △Ct _experimental group_ - △Ct _control group_, △Ct = Ct (target gene) - Ct (internal reference).

**Table 2:** Primer sequence.

**Gene**	**Primer sequence**
MALAT1	F: 5′- CTCACTAAAGGCACCGAAGG-3′
	R: 5′- GGCAGAGAAGTTGCTTGTGG-3′
Notch-1	F: 5′- CACTTGGCTGCCCGATACTCT-3′
	R: 5′- GCCCATGTTGTCCTGGATGTT-3′
Collagen-I	F: 5′- GAGGGCCAAGACGAAGACATC-3′
	R: 5′- CAGATCACGTCATCGCACAAC-3′
Collagen-III	F: 5′- GGAGCTGGCTACTTCTCGC-3′
	R: 5′- GGGAACATCCTCCTTCAACAG-3′
Fibronectin	F: 5′- ACAACCAGAGGAGGCACAAG-3′
	R: 5′- CCGTGTAAGGGTCAAAGCAT-3′
GAPDH	F: 5′- ACGGCAAGTTCAACGGCACAG-3′
	R: 5′- GACGCCAGTAGACTCCACGACA-3′

Note: F, forward; R, reverse; MALAT1, metastasis-associated lung adenocarcinoma transcript 1; GAPDH, glyceraldehyde phosphate dehydrogenase.

### Western blot analysis

Thoracic aorta homogenate was prepared and total protein content of thoracic aorta was evaluated according to the instruction of bicinchoninic acid kit (Pierce, Rockford, IL, USA). Degenerated proteins were mixed with loading buffer, boiled at 95°C for 10 min, and speared with 10% polyacrylamide gel electrophoresis and transferred onto polyvinylidene fluoride membrane, then sealed with 5% bovine serum albumin (BSA) for 1 h, added with primary antibodies Notch-1 (diluted at 1: 1000, Cell Signaling Technology, Boston, MA, USA), collagen-I (diluted at 1: 500, Novus Biologicals, Littleton, CO, USA), collagen-III (diluted at 1: 500, Novus Biologicals, Littleton, CO, USA), Fibronectin (diluted at 1: 500, Abcam, Cambridge, MA, USA), Bax (diluted at 1: 1000, Cell Signaling Technology, Boston, MA, USA), Bcl-2 (diluted at 1: 1000, Cell Signaling Technology, Boston, MA, USA) and GAPDH (diluted at 1: 1000, Cell Signaling Technology, Boston, MA, USA), incubated at 4°C for one night, rinsed by tris buffer solution with tween (TBST), 3 times/5 min and incubated by relative secondary antibody (Abcam, Cambridge, MA, USA) for 1 h. Next, the membrane was added with enhanced chemiluminescent regent (ECL), exposed in darkroom, and GAPDH was used as internal reference. After photography analyzed by Bio-Rad Image Lab system (GEL DOC EZ IMAGER, Bio-rad, CA, USA), the target band was conducted with grey value analysis by Image J software.

### Statistical analysis

All data were analyzed by SPSS 21.0 software (SPSS, Inc., Chicago, IL, USA). The measurement data were expressed as mean ± standard deviation, comparisons between two groups were conducted by *t*-test, while comparisons among multiple groups were assessed by one-way analysis of variance (ANOVA), and Tukey’s post hoc test was use for pairwise comparisons. The receiver operating characteristic curve (ROC curve) was drew and the diagnostic efficiency of MALAT1 expression to HTN patients was measured by area under the curve (AUC). *p* value < 0.05 was indicative of statistically significant difference.
